# Tugging at the Heart Strings: The Septin Cytoskeleton in Heart Development and Disease

**DOI:** 10.3390/jcdd7010003

**Published:** 2020-01-09

**Authors:** Kelsey Moore, Reece Moore, Christina Wang, Russell A. Norris

**Affiliations:** Cardiovascular Developmental Biology Center, Department of Regenerative Medicine and Cell Biology, College of Medicine, Children’s Research Institute, Medical University of South Carolina, 171 Ashley Avenue, Charleston, SC 29425, USA; moorkels@musc.edu (K.M.); moorere@musc.edu (R.M.); wangch@musc.edu (C.W.)

**Keywords:** septin cytoskseleton, primary cilia, binucleation, septin rings, cardiac septins, actin, microtubules

## Abstract

Septin genes were originally identified in budding yeast in 1971. Since their original discovery, at least 13 mammalian genes have now been found, which give rise to a vast array of alternatively spliced proteins that display unique spatial-temporal function across organs systems. Septin’s are now recognized as the 4th major component of the cytoskeleton. Their role in regulating ciliogenesis, actin and microtubule organization and their involvement in mechanotransduction clearly solidify their place as both a responder and driver of cellular activity. Although work on septin’s has escalated over the past decades, knowledge of septin function in the heart remains rudimentary. Whereas many cardiovascular diseases have been associated with genetic loci that include septin genes, new and additional concerted efforts will likely uncover previously unrecognized mechanisms by which the septin class of proteins contribute to clinical cardiac phenotypes. In this review, we place known function of septin proteins in the context of heart development and disease and provide perspectives on how increased knowledge of these proteins can mechanistically inform cardiac pathologies.

## 1. Introduction

Discovered in 1971, septins are highly conserved GTP-binding and self-assembling filamentous proteins [1,2]. Through cell division screens using the budding yeast *Saccharomyces cerevisiae*, Hartwell was the first to identify septins as essential genes for cytokinesis and cell polarity [2,3]. In the 40 years since, septins have been identified in many other organisms, including *Drosophila*, mice and humans, and have been implicated in a plethora of developmental and disease processes. Emerging as important regulators of the cytoskeleton, pathways that rely on septins present exciting avenues across various fields. The role of septins as building blocks during cardiac development and their function in cardiac pathologies remains poorly understood. Therefore, in this review, we have summarized the knowledge of septin biology thus far and provided perspectives on their involvement in various developmental and acquired cardiac diseases.

## 2. Septins at a Glance

Only two septin genes have been identified in *Caenorhabditis elegans* (*Unc-59* and *Unc-61*), whereas 13 members are present in humans (septins 1–12 and 14) [4]. Alternative splicing further complicates the “septome” in mammals; in humans, for example, the shuffling of exons at the N- and C- terminus of SEPT9 results in an impressive 15 protein isoforms with unique functions [5,6]. Together, the isoforms make up the “septin code.”

Each septin isoform contains a guanine nucleotide binding domain and shares structural similarities with the P-loop GTPase and ATPase superfamily [7]. An upstream septin-unique element (SUE) and slow GTP hydrolytics, however, differentiate septins from conventional kinesins, myosins, and Ras proteins. Additional conserved elements include a polybasic region in the N-terminus that interacts with membrane lipids [8] and an extension of coiled-coil domains in the C-terminus [4]. Septin isoforms are distinguished by N- and C-terminus length and sequence differences, which result in their unique functions. The amount of coiled-coil domains following the GTPase region allows the classification of septins into four groups: two domains exist in the septin-2 group (SEPT1, 2, 4, and 5), zero in septin-3 (SEPT3, 9, and 12), one in septin-6 (SEPT6, 8, 10, 11, and 14), and one in septin-7 (SEPT7). These coiled-coil domains and their structural positioning contribute to the interactions between septins and other cytoplasmic substrates [8] and, as a result, their formation of highly ordered filamentous structures.

## 3. Assembly of Septin Filaments

Diverse oligomer and filament structures result from the dynamic interactions between septin monomers. Although their distinct mechanisms are unknown, GTP-binding and the TRiC/CCT chaperonin facilitate the dimerization of septin monomers to form canonical and non-canonical complexes [9]. Canonical complexes, such as SEPT2/6/7/9 and SEPT3/5/7 [10,11], are composed of septin monomers from different septin groups. Conversely, non-canonical complexes, such as SEPT4/5/8 [12], are formed from substrates of the same septin group and do not copy the typical SEPT2/6/7/9 oligomer arrangement. Both types of heteromers can polymerize into non-polar filaments via end-to-end contact and the binding of coiled-coil domains in their subunits’ C-terminals [13]. The pairing of filaments via end-end joining and/or lateral interactions results in the formation of structures including linear bundles, gauzes and circular rings [4]. Although many septin structures have been observed in vivo and manipulated in vitro, much remains to be discovered regarding the regulation of filament polymerization and about their interactors.

Oligomer and polymer assembly is not only regulated by septin protein structure, but also by posttranslational modifications and interactions with binding proteins. In addition to septin–septin mediated conformational changes, posttranslational modifications can affect the folding and binding of septin dimers. SUMOylation, acetylation and phosphorylation events were illustrated in a recent review, which highlighted their effects on filament assembly and function during specific cell cycle and developmental stages [14]. An additional layer of filament regulation is provided by the identification of septin-binding partners. For instance, Joberty, Perlungher and colleagues showed that BORG3 binds septin and inhibits assembly in the absence of Cdc42 [15]. Later, this interaction was linked to cardiac developmental defects [16] and mechanotransduction pathways of cancer associated fibroblasts [17]. Several other binding partners are involved in the septin–cytoskeleton networks that are discussed in the remainder of this review.

It is increasingly understood that septins are important regulators of the cytoskeleton through direct interactions with actin and microtubule proteins. Several excellent reviews discuss the ability of septin to directly bind and mediate actin and microtubule filament organization, and the effect on a plethora of molecular and cellular processes [4,18,19,20,21,22]. In particular, Spiliotis addresses the less understood mechanisms of actin and microtubule dynamics, including how network positions are spatially and temporally controlled by septin networks [22]. It is also important to note that, in turn, the cytoskeleton is able to regulate septin filaments. Of the established functions of septin–cytoskeletal networks and cross-talk, many have emerged in critical developmental processes. However, we will limit our discussion to themes involving cardiac development and disease, and present our perspectives on the vastly understudied role of septins in the heart.

## 4. Septins in the Heart

Increasing evidence supports a role for septin proteins in the regulation of microtubule, actin and integrin proteins, which are essential to multiple facets of normal heart development. Through unknown mechanisms, embryonic lethality in *Sept7-*, *Sept9-*, and *Sept11*-deficient mice suggests that septins are essential for normal embryogenesis [23,24]. To date, Septins 2 and 5–11 have been observed within developing and adult zebrafish, mouse and human hearts [25,26,27]. Several of these septins have been shown to regulate the organization of microtubule-based cilia, actin and integrins, all of which are involved in essential processes of cardiac development and disease.

## 5. Cilia–Septin Interactions

Microtubule-based cilia are indispensable for normal heart and valve development and have been shown to be regulated by septins. Cilia, specifically non-motile primary cilia, were first identified in nonmitotic cardiomyocytes in 1969 via electron microscopy of chick, rabbit, mouse, and lizard embryonic hearts [28]. Since then, cilia have been found throughout the mammalian embryonic heart tube, the atria and trabeculated myocardium [29], in the endocardium and endocardially derived mesenchymal cells [30,31,32,33], and in the epicardium [34]. By adulthood, however, much less is known about cell-types that express primary cilia. Two recent reports have demonstrated that they are persistent on cardiac fibroblasts yet absent on most other cell types [31,35], suggesting they likely play a role during development and in adult ventricular homeostasis. Cilia defects during embryogenesis have been shown to disrupt the left–right asymmetry and result in congenital heart defects [36]. Additionally, loss of cilia was shown to result in abnormalities in aortic and mitral valve architecture and contributed to mitral valve prolapse disease phenotypes in humans and mice [31,32,33]. The latter findings were based on mutations discovered in patients and support the necessity for cilia in cardiovascular development. However, the distinct intracellular mechanisms mediated by cilia are undefined, and a role for septins has not yet fully emerged.

It is known that various ciliary signaling pathways are essential for cardiac development, including TGF-beta, Wnt, hedgehog and BMP mechanisms, as well as cell polarity factors [36]. Of these, hedgehog signaling was lost in SEPT2, 6, or 7 deficient animals [27,37]. As hedgehog signaling contributes to multiple signaling pathways, the disruption of it could lead to a broad range of phenotypes. In addition, septin complexes have been identified at the base of cilia and were shown to act as a diffusion barrier [38]. As such, it is possible that septins could mediate signal transduction and ciliary receptor expression during cardiovascular development. Furthermore, a planar cell polarity component, *Fritz*, has been shown to localize with cilia and ciliary septins [27]. Since cardiac morphogenesis relies on highly coordinated cell migration and spatial organization, defects in cell polarity mediated by ciliary septins may have severe effects on embryogenesis and should be investigated further.

In addition to ciliary signaling efficiency, structure and length potentiate cilia function. This was first supported by cilia-dependent cell cycle experiments showing that suppression of primary cilia axonemal growth limits transitions from G0/G1 to S phase [39]. Since septins are able to bind and alter the activity of microtubule-stabilizing protein MAP4, it is not surprising that many reports have shown septins localized to the ciliary axoneme and are able to regulate ciliogenesis [40]. Proteomic and proximity ligation experiments support the axonemal distribution of septin proteins [41] and co-localization has already been observed in multiple cell types and animal models (see [4] for a detailed review). For example, Ghossoub and colleagues showed that SEPT2, 7 and 9 co-localize with the axoneme and that dismantling this complex via siRNAs inhibited ciliogenesis in kidney epithelial cells [42]. In another study, *sept7b* knockdown resulted in defects in ciliogenesis and left–right asymmetry in zebrafish hearts [43]. This is the first study to directly test septin manipulation in heart embryogenesis and would benefit from validation in mammalian models. Taken together, these data support a role for septins in ciliogenesis and ciliary signaling, which are essential for the development of highly complex organ systems including the heart.

## 6. Actin–Septin Networks

A role for septins in cardiac development is also presented by their ability to regulate actin organization. Since their identification, septins have been reported to co-localize with the actin cytoskeleton and most prominently, actin stress fibers [43]. Recent studies report the ability of septins to polymerize and control the morphology of actin filaments in vitro [44]. Septin-9 (SEPT9), in particular, is able to crosslink and bundle actin monomers independently of other septins using three distinct modes of actin-binding [45]. SEPT6 and SEPT7 were also shown to promote actin polymerization through interactions with Arp2/3 and cortactin [38]. These septin proteins (6, 7, and 9), along with septin-2 (SEPT2), form a complex that is highly expressed in embryonic cardiac cells and diminishes by adulthood [38]. This suggests that septin–actin networks are developmentally regulated in the heart. Lastly, since actin monomers are able to translocate into the nucleus and regulate RNA polymerases and various transcription factors [46], septin-mediated changes in actin–filament organization may affect many downstream processes.

Why is the actin cytoskeleton important for cardiovascular development and how could septins play a role in this? First, a large portion of heart cells are derived from at least one epithelial to mesenchyme transition (EMT), a process that is stimulated by de-stabilization of adherens junctions and dynamic processes of actin remodeling [47,48]. Defects in various EMT pathways have been shown to affect valve morphogenesis [49] and EMT is re-activated in valve disease [50,51,52] and cardiac fibrosis [53]. Actin network stabilization by septins may stabilize normal EMTs during cardiac development and could be targeted in disease progression.

Second, cardiomyocyte cell division is orchestrated by actin and microtubule dynamics. Humans are born with roughly 1 billion cardiomyocytes which accumulate to 4 billion by 20 years, when proliferation ceases [54]. The cessation of cytokinesis following birth leads to mononucleated polyploid or binucleated cardiomyocytes and is a highly researched topic for the development of regenerative therapeutics. A wide range of genes have been implicated in this process and include cell cycle regulators, signal transducers, epigenetic modifiers, and mitochondria metabolism. Overall, it is accepted that the inability to form an actomyosin contractile ring leads to incomplete furrow ingression and abscission failure, resulting in binucleated cells. Recently, it was shown that binucleation occurs as a result of supernumerary centrosomes and/or unpaired centrioles [55]. Given the historical role of septins as aids in cytokinetic machinery assembly [2], it is possible that one or more of the septin proteins could be involved in cardiac-specific cytokinesis. Ahuja and colleagues were the first to show the developmentally regulated protein expression of septins 2, 6, 7, and 9 in the heart, which diminishes following birth [25]. Each of the septins localized to the cleavage furrow in dividing cardiomyocytes in vitro and septin-2 localization was shown to be dependent on actin and myosin localization. Also, it has been observed that loss of septin-7 in zebrafish leads to abnormal heart morphology as a result of disrupted myofibrils and contractile abilities [43]. Recent reports have shown that deletion of SEPT2, SEPT7, and SEPT11 causes defects in early stages of cytokinesis and ultimately results in binucleation. In addition, SEPT9 loss causes defects in the cytokinesis, which is critical for midbody abscission, the final stage that results in the separation of daughter cells [5]. Septin proteins in groups 3, 6, and 7 are additionally regulated by SUMOylation during the cell cycle to modulate filament bundles and cytokinesis [19,56]. Thus, in the absence of septins or SUMOylation, cells undergo karyokinesis without cytokinesis, very similar to what is observed in postnatal cardiomyocytes. Although correlative, it is interesting to speculate that loss of the “septin code” after birth may provide a molecular explanation for why myocytes stop dividing. If true, the re-expression of critical septins involved in abscission may reignite completion of cytokinesis and provide a missing key needed to enhance cardiomyocyte regrowth with potential for regeneration.

Third, actin filaments are essential for cardiomyocyte contractility. Myofibrils, which develop during embryogenesis, are a well-characterized structure of actin, myosin, titin, and other binding proteins. It is appreciated that cardiomyopathies result from defects in intercalated discs, the cell–cell adhesion complex between cardiomyocytes, and increased stiffness of the sarcomere [57]. However, recent reports have identified mutations in actin-interacting proteins that can cause inheritable cardiomyopathies. In particular, actin assembly factor, Arp2/3, enhances the energetically unfavorable process of filamentous-actin (F-actin) polymerization from globular-actin (G-actin) monomers [57]. Of importance to our discussion, Arp2/3 localizes to the membrane in response to small GTPase recruitment of actin filaments to the membrane [58]. This process works in synergy with profilin and is inhibited by cofilin [59]. The following evidence supports the function of septins in this process: i) SEPT2, 6, 7, and 9 were shown to be expressed in cardiomyocytes during timepoints of myofibril development [25]; ii) septins, which are P-loop GTPases, interact with the membrane via a phosphoinositide binding domain located in the N-terminus [8]; iii) SEPT6 binds to Arp2/3 branched actin filaments [38] and; iv) SEPT9 stabilizes actin filaments from depolymerization by cofilin and myosin [45]. Overall, these data suggest that septins, specifically SEPT2, 6, 7, and 9, may stabilize actin polymerization at the membrane during cardiomyocyte development and contribute to disease inception. Mutations in septins or septin interactors should be explored in these pathways.

## 7. ECM-Integrin-Septins

Septins have also been shown to regulate integrins and as such, provide a link to fibrotic pathways that are involved in cardiac disease. Integrin proteins attach the cell to the extracellular matrix (ECM) to form focal adhesion (FA) complexes and, as such, provide an ECM–intracellular connection. FAs are important for cellular processes including migration through ECM substances, mechanotransduction and fibrotic events. Spiliotis and colleagues showed that FA maturation is regulated by septin–actin networks and, more specifically, that SEPT9 promotes the migration of renal epithelial cells during EMT [18]. These data support a potential role for SEPT9 in regulating EMT during cardiac morphogenesis (as described above). In terms of fibrosis, defects in or loss of septins could affect the migration of cardiac fibroblasts and, by affecting integrins, alter the ECM remodeling and mechanotransduction of cardiac mesenchymal cells. For example, left ventricular fibrosis was more prevalent in patients with mitral regurgitation with valve prolapse versus without [60]. It is possible that ECM production is upregulated in MVP patients in response to severe mechanical stress cues received by focal adhesions. However, the molecular pathways governing integrin responses and how septins regulate them are unknown.

The detection of extracellular forces and ECM composition by the cell’s focal adhesion complexes is an essential facet of cellular development and organogenesis. This ability has been shown to affect the development of multiple cardiac structures. Of such, normal growth of the myocardium occurs in concordance with the production of ECM by cardiac fibroblasts in a feed-forward mechanism and contributes to the contractile ability of cardiomyocytes [61]. The compaction, elongation, and deposition of the ECM, as mediated in part by Rho-GTPases, are integral to mitral valve morphogenesis [62]. It is likely that septins cooperate in these important pathways, as they have been shown to respond to changes in the extracellular matrix and mechanical cues. In particular, septin-9 was upregulated in endothelial cells grown in low-stiffness compared to high-stiffness environments. Additionally, loss of septin-9 lead to actin filament redistribution from the periphery to the cortex, as well as observed increases in RhoA production [63]. It is also known that both the presence of septin proteins and their interactions with small GTPases are required for proper remodeling of the ECM and ECM deposition in fibroblasts [17]. As septins are increasingly recognized as regulators of actin organization and resultant stress fiber and focal adhesion formation, it is intuitive to further explore their role in sensing mechanical cues received by the extracellular environment, especially in the heart.

Signaling through TGF-beta is known to regulate cardiac development and fibrosis and can be activated by integrin interactions. More specifically, TGF-beta promotes the activation of mesenchyme cells to cardiac fibroblasts and, as a result, increases ECM production [64,65]. Fibroblast activation through TGF-beta also coincides with alpha-smooth muscle actin expression. Although controversial, alpha-smooth muscle actin is considered a read-out for fibroblast to myofibroblast transition [66]. Septin proteins have been identified in TGF-beta-dependent fibrotic pathways. The presence of either septin-4 (SEPT4) and septin-9 (SEPT9) was shown to attenuate liver fibrosis by decreasing TGF-beta, alpha-smooth muscle actin, and collagen I production in vivo [67,68]. Septin-2 (SEPT2) protein expression also alleviated alpha-smooth muscle actin expression and decreased motility in adventitial fibroblasts [69]. Of clinical importance, septin-2 protein expression was decreased in human mitral heart valves with myxomatous degeneration [70]. Together, these data provide insights into a septin-2 mediated anti-fibrotic response. On the other hand, septin-6 (SEPT6) promoted liver fibrosis through TGF-beta and MAP-kinase pathways [71]. It is unknown whether the other 10 septin proteins are involved in fibrosis. However, actin–septin networks were broadly identified to promote the activation of cancer-associated fibroblasts [17]. Further studies are necessary to define the precise functions of septins in cardiac fibrosis.

## 8. Conclusions

In the 40 years of research following Hartwell’s discovery, septins were identified in many eukaryotic organisms, and we now understand the evolution of septin genes and their encoded proteins. Impressive structural and biochemical studies have uncovered many aspects of the septin interactome and have established the “septin code.” As a result of their diverse interactions with actin, microtubules, integrins and other cytoskeleton-binding proteins, septins are now considered the fourth component of the cytoskeleton. They are increasingly recognized in important cellular and molecular pathways, and thus present ample opportunities for future investigation. The molecular functions of septins have been identified in various cellular pathways that are essential for development and disease progression, many of which are critical for normal heart morphogenesis and disease inception. Many heart diseases have a genetic and, thus, a developmental basis, and carry a significant burden of morbidity and mortality. Thus, it is important that future studies acknowledge and consider the potential functions of septin genes and proteins in an effort to advance therapeutics for a broad range of patients with cardiovascular diseases. Indeed, based on a wide variety of cardiovascular diseases associated with the genetic positions of many septin genes, it is likely that this family of genes will play a direct and relevant role in the etiology and pathogenesis of human heart diseases (Table 1).

Surprisingly, research on septins in the heart is still in its infancy, with a very limited number of reports describing their function. Testing whether septin function in the heart is conserved across tissues may provide key insight into mechanisms underlying both common and rare cardiovascular diseases, and will likely be the focus of concerted scientific efforts in the future.

## Figures and Tables

**Table 1:** Cardiovascular diseases associated with the genetic positions of many septin genes.

Human Chromosome Position	Gene Size (Base Pairs)	Potential Cardiovascular Disease Association	OMIM Reference ID
16p11.2	17,859	Mitral Valve Prolapse	15770
2q37.3	38,928	None Described	
17q22	20,569	Hpertension	145500
22q11.21	10,309	Cat Eye Syndrome, CATCH22, DiGeorge Syndrome, Emanuel Syndrome	115470, 125520, 611867, 608363, 18840, 609029
22q13.2	21,950	None Described	
17q25.3	220,028	Hypertension	603918
16p13.3	10,853	16p13.3 Deletion Syndrome, 16p13.3 Duplication Syndrome	610543, 613458
Xq24	77,647	None Described	
5q31.1	56,425	None Described	
2q13	71,408	None Described	
4q21	90,682	None Described	
7p11.2	69,246	None Described	
7p14.2	104,376	Ventricular Tachcardia, Telangiectasia	614021, 610655
